# Integrated transcriptomic and proteomic profiling in keloid tissue

**DOI:** 10.7717/peerj.21407

**Published:** 2026-06-24

**Authors:** Haitao Lu, Yunhua Zhao, Baoqiang Li, Yinhui Yao, Lijun Liu, Liyuan Liu, Jiali Zhang, Lei He, Xinsuo Duan

**Affiliations:** 1 Department of Dermatology, The Affiliated Hospital of Chengde Medical University, Chengde, China; 2 Department of Otolaryngology, Chengde Central Hospital, Chengde, China

**Keywords:** Keloid, Multi-omics integration, PI3K-AKT pathway, Extracellular matrix, Epidermal barrier, Proteomics

## Abstract

**Background:**

Keloid is a pathological skin fibrosis disease characterized by abnormal proliferation of dermal fibroblasts and excessive deposition of extracellular matrix (ECM). Its high recurrence rate necessitates an in-depth investigation of molecular mechanisms to develop effective treatment strategies.

**Methods:**

In this study, 20 samples of keloid tissue and adjacent normal skin were collected. Transcriptome sequencing and proteome analysis were performed, combined with differential expression analysis, functional enrichment, weighted gene co-expression network analysis (WGCNA), and protein interaction network analysis using the STRING platform, to systematically explore the molecular characteristics of keloid.

**Results:**

A total of 4,994 genes and 828 proteins were found to be differentially expressed in keloid tissue, with enrichment primarily observed in the PI3K-AKT, TGF-β, ECM-receptor interaction, and mitogen-activated protein kinase (MAPK) signaling pathways. Upregulated hub genes MAGED1, FN1, and COL5A2, as well as downregulated hub genes including IL20RA and CLDN4, were identified by WGCNA. Their respective interaction networks were associated with excessive ECM accumulation and disruption of epidermal structure.

**Conclusion:**

This study has confirmed that the regulatory network of epidermal dysfunction caused by excessive activation of fibroblasts in keloids through multi-omics integration, which provides a theoretical basis for multi-target therapy targeting ECM remodeling and PI3K-AKT/MAPK pathway.

## Introduction

Keloid is a pathological skin fibrosis disease with abnormal proliferation of dermal fibroblasts and excessive deposition of extracellular matrix (ECM) induced by trauma, inflammation, or genetic factors. Clinically, keloids typically present as invasive lesions that extend beyond the boundaries of the original injury and are frequently accompanied by pruritus, pain, and joint dysfunction, significantly impairing patients’ quality of life (Jagdeo, Kerby & Glass, 2021; Murray, Pollack & Pinnell, 1981). Although treatments such as surgical excision, pharmacological intervention, and radiotherapy can provide temporary symptomatic relief (Mustoe et al., 2002), recurrence rates exceed 50% (Shook et al., 2018). Therefore, a deeper investigation into the molecular mechanisms of keloid formation is crucial for the development of effective therapeutic strategies.

Recent research has revealed that the pathological process of keloid is closely associated with the dysregulation of several critical signaling pathways. As a key biological process, the abnormal remodeling of ECM leads to excessive deposition of a large amount of collagen and other components, which is the typical pathological feature of keloid (Shook et al., 2018). Cellular REDOX dysregulation is also closely related to the fibrotic process of keloids. Studies have shown that reactive oxygen species (ROS) are significantly increased in keloid patients, which not only directly damage mitochondrial function, but also activate proinflammatory pathways such as NF-κB, induce abnormal proliferation of fibroblasts and ECM synthesis, and aggravate oxidative damage of scar tissue (Wang et al., 2021). Furthermore, signaling cascades such as transforming growth factor-β (TGF-β), phosphatidylinositol 3-kinase/protein kinase B (PI3K-AKT), and Janus kinase/signal transducer and activator of transcription (JAK-STAT) are key regulators of fibroblast proliferation, migration, and ECM metabolism (Lacombe & Morice-Picard, 2014). For instance, the PI3K-Akt pathway, a major regulator of cell survival and proliferation, promotes fibroblast activation by stimulating downstream mTOR or inhibiting GSK-3β; its overactivation can lead to uncontrolled fibroblast proliferation (Liu et al., 2024). Similarly, ECM-receptor interactions, such as integrin-mediated focal adhesion signaling, can also enhance cell-matrix adhesion through FAK/PI3K-AKT axis, further exacerbating ECM deposition (Huang et al., 2023). TGF-β pathway can phosphorylate Smad proteins to directly up-regulate the expression of genes such as COL1A1 and FN1, and form a positive feedback loop with PI3K-Akt pathway to synergistically drive fibrosis (Zhang et al., 2020). However, although the individual roles of these pathways have been partially elucidated, their interaction and synergy in keloid and the dynamic regulatory nodes at the multi-omics level still need to be further explored.

With the rapid development of bioinformatics, the collaborative application of multi-omics technology provides a new and hierarchical research perspective for systematically revealing the molecular mechanism of complex diseases (Baysoy et al., 2023). Especially in the study of keloid, the combined analysis of transcriptomics and proteomics is particularly critical. Transcriptomics can dynamically reflect the “upstream signal” of gene transcription regulation through mRNA expression profile. For example, Deng et al. (2021) study have shown significant upregulation of ECM-related genes such as COL5A1 and THBS2 at the mRNA level in keloid tissue, indicating transcriptional activation of ECM synthesis. However, high mRNA expression is not directly equivalent to efficient production of functional proteins. Combined with proteomics, the actual amount of COL5A1 protein deposition and post-translational modification analysis (such as collagen hydroxylation and glycosylation) could be accurately captured, and the biological effects of “downstream function execution” of ECM over deposition could be further verified (Liu et al., 2022b). The integration of transcriptomics and proteomics constructs a bi-directionally validated molecular regulatory network along the “transcription-translation” axis (Kreitmaier, Katsoula & Zeggini, 2023).

In addition, weighted gene co-expression network analysis (WGCNA) analysis based on transcriptome can accurately screen out the clinical hub genes, and then verify the interaction network of their coding proteins through the proteome data to identify the key protein-protein interaction (PPI) modules (Vogel & Marcotte, 2012). This multi-dimensional association analysis can not only break through the interference of single transcriptomes which are susceptible to RNA degradation, alternative splicing and other factors, but also overcome the detection sensitivity of proteomics which is limited by low abundance proteins and significantly improve the reliability of data.

Based on transcriptome sequencing data and proteomic analysis, this study systematically screened differentially expressed genes (DEGs) and proteins (DEPs) between keloid and normal skin tissue. Through functional enrichment analysis, PI3K-AKT, extracellular matrix-receptor interaction, TGF-β and JAK-STAT pathways were identified as the core regulatory network. The key genes and key protein interaction networks of keloid were further identified. This study provides a new perspective for in-depth analysis of the pathological mechanism of keloid, which has important translational value.

## Materials and Methods

### Sample collection

Keloid and adjacent normal skin tissue samples (the latter taken 1 cm from the visible margin of the keloid lesion) were collected from patients who underwent surgical resection at affiliated hospital of Chengde Medical University between 1 October 2024 and 1 April 2025. Each sample was obtained from a distinct patient (no individual contributed more than one biopsy), giving 20 biological replicates per group and ensuring that only biological variability is represented. Samples were immediately flash-frozen in liquid nitrogen and stored at −80 °C until analysis.

All participants provided written informed consent, and the study was approved by the Ethics Committee of Affiliated Hospital of Chengde Medical University (approval no. CYFYLL2023520). Basic demographic data were recorded, and keloid severity was assessed with the modified Vancouver Scar Scale (pigmentation, vascularity, pliability, pain/pruritus).

Patients younger than 18 or older than 60 years, or those receiving systemic treatments that could interfere with transcription (corticosteroids, 5-fluorouracil, radiotherapy), were excluded. RNA sequencing was performed at 40 million reads per sample, a depth that exceeds the 5/CV^2^ threshold; consequently, no technical replicates were generated.

The raw data in this study have uploaded to the China National Center for Bioinformation, can be accessed at https://ngdc.cncb.ac.cn/omix/release/OMIX012709.

### Transcriptome sequencing and analysis

Transcriptomic profiling was conducted by Majorbio Bio-Pharm Technology Co., Ltd. (Shanghai, China) using the Illumina NovaSeq 6000 platform. Total RNA was extracted from keloid and normal skin tissues using TRIzol reagent (Invitrogen, Carlsbad, CA, USA), and RNA integrity was assessed with an Agilent 2100 Bioanalyzer (Agilent Technologies, Santa Clara, CA, USA). Only samples with RNA Integrity Number (RIN) >7.0 were included. Polyadenylated mRNA was enriched *via* oligo (dT) magnetic beads (Thermo Fisher Scientific, Waltham, MA, USA), and strand-specific cDNA libraries were prepared using the NEBNext Ultra^TM^ RNA Library Prep Kit for Illumina (New England Biolabs, Ipswich, MA, USA). Libraries were sequenced in paired-end mode (150 bp reads) with at least 40 million reads per sample. Raw sequencing data were quality-checked with FastQC (v0.11.9) (https://github.com/OpenGene/fastp), adapters and low-quality sequences (Phred score < 20) were removed *via* Trimmomatic (v0.39) (https://github.com/usadellab/Trimmomatic/). High-quality reads were aligned to the human reference genome (GRCh38) using HISAT2 (v2.2.1) (https://github.com/DaehwanKimLab/hisat2) with default parameters. Transcript abundance was quantified using StringTie (v2.1.4) and normalized as transcripts per million (TPM) for subsequent differential expression analysis.

The statistical power for transcriptome samples was estimated using the RNASeqPower (v1.44.0, https://bioconductor.org/packages/RNASeqPower/) with parameters set to effect = 2.0 and alpha = 0.05. RNASeqPower was 0.80. With 20 samples per group, the analysis yielded a power of 0.80, meaning there is an 80% probability of correctly detecting a true positive result (*i.e*., a gene with at least a 2-fold change at α = 0.05).

### Differential expression analysis

DEGs between keloid and normal skin groups were identified using DESeq2 (http://bioconductor.org/packages/stats/bioc/DESeq2/). Raw read counts were normalized using the median-of-ratios method, and dispersion was estimated through a negative binomial model. Genes with |log2 (*fold change*)| > 1 and adjusted *P*-value (Benjamini-Hochberg) <0.05 were classified as statistically significant (Love, Huber & Anders, 2014). Volcano plots were generated using the ggplot2 package (v3.4.0) (Wickham, 2016) to visualize log2 fold changes against adjusted *P*-values. Hierarchical clustering of DEGs was displayed as heatmaps using the heatmap package (v1.0.12), with row-wise Z-score normalization to emphasize expression patterns.

### Gene enrichment analysis

Functional annotation of DEGs was conducted using Gene Ontology (GO) and Kyoto Encyclopedia of Genes and Genomes (KEGG) (Kanehisa et al., 2025; Kanehisa, 2019; Kanehisa & Goto, 2000) databases *via* the cluster Profiler package (v4.4.4). As per the citation guidelines: GO (http://geneontology.org/), KEGG (https://www.genome.jp/kegg/) (Kanehisa et al., 2025; Kanehisa, 2019; Kanehisa & Goto, 2000). Enrichment analysis was conducted using Goatools (https://github.com/tanghaibao/GOatools) with a significance threshold of adjusted *P*-value < 0.05. GO terms were categorized into biological processes (BP), molecular functions (MF), and cellular components (CC). Gene Set Enrichment Analysis (GSEA) (v4.3.2) was employed to identify pathway-level dysregulation, using the Molecular Signatures Database (MSigDB) Hallmark gene sets. Pathways with |normalized enrichment score (NES)| > 1.5 and false discovery rate (FDR)-adjusted *P*-value < 0.05 were considered significantly enriched.

### Proteomics sequencing and analysis

Transcriptome analysis was performed by Majorbio Bio-Pharm Technology Co., Ltd. (Shanghai, China) using a timsTOF Pro2 mass spectrometer. Proteins were extracted from tissues using Radio Immunoprecipitation Assay (RIPA) lysis buffer (Thermo Fisher Scientific, Waltham, MA, USA) supplemented with protease inhibitors (Roche, Switzerland). Protein concentration was determined *via* BCA assay (Pierce, USA), and 100 μg of protein per sample was digested with sequencing-grade trypsin (Promega, USA) at 37 °C for 16 h. Tryptic peptides were labeled with TMT 10-plex isobaric tags (Thermo Fisher Scientific) following manufacturer protocols. Labeled peptides were fractionated using a nanoLC system (UltiMate 3000; Thermo Fisher Scientific) with a C18 column (75 μm × 25 cm, 2 μm particles) and analyzed in data-dependent acquisition (DDA) mode. MS raw files were processed using MaxQuant (v2.1.0) against the UniProt human reference proteome (release 2025_03). Search parameters included a precursor mass tolerance of 20 ppm, fragment mass tolerance of 0.5 Da, and a maximum of two missed cleavages. Proteins with ≥2 unique peptides, a false discovery rate (FDR) <1%, and |log2(*fold change*)| > 1 with *P*-value < 0.05 (Student’s t-test) were classified as DEPs.

### Hub gene identification

WGCNA (v1.71) was implemented to identify hub genes associated with keloid pathogenesis (Langfelder & Horvath, 2008). A signed hybrid network was constructed using all DEGs. The soft thresholding power (β = 20) was selected based on scale-free topology criterion (R² > 0.8). A topological overlap matrix (TOM) was generated to measure pairwise gene connectivity, and modules were detected *via* dynamic tree cutting (minimum module size = 30, merge Cut Height = 0.25). We designated these modules by assigning distinct colors (‘turquoise’, ‘blue’, ‘grey’). Module eigengenes (MEs) were calculated as the first principal component of each module. Module-trait associations were quantified using Pearson correlation coefficients between MEs and clinical traits (keloid *vs*. normal), with significance determined by *P*-values adjusted *via* Bonferroni correction. Genes within the most significant modules (|correlation coefficient| > 0.8, *P* < 0.001) were ranked by intramodular connectivity (kME). The top 10 genes per module with the highest kME, which measures the correlation between a gene and the module eigengene, were designated as hub genes.

### Protein interaction network analysis

PPI network was established using STRING database v11.0 (https://cn.string-db.org/) (Szklarczyk et al., 2019) and network modeling method. A confidence score ≥0.4 was set as the threshold to construct the PPI network, which represents a medium confidence level to balance the inclusion of meaningful interactions and the reduction of false positives. The confidence of each interaction in the STRING database is quantified by a Combined Score, which integrates various evidence channels such as gene co-expression, experimental data, and curated database knowledge. A higher combined score indicates a more reliable protein-protein association.

## Results

### Patient characteristics

**Figure 1:**
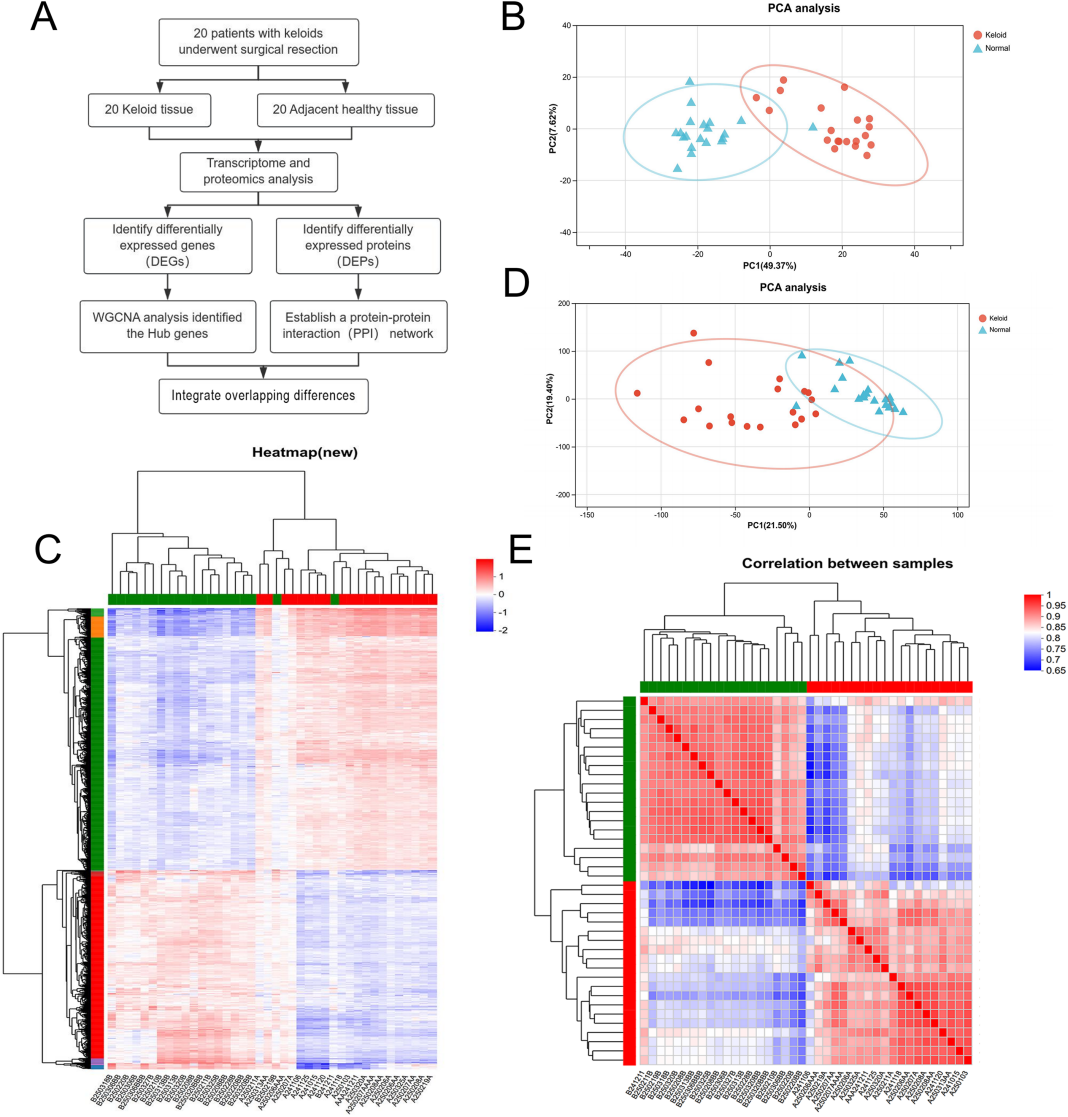
Data quality assessment. (A) Serials selection process. (B) Transcriptome principal component analysis plots of keloid *vs*. normal skin groups. The horizontal and vertical axes represent the first principal component (PC1) and the second principal component (PC2), respectively, with each point representing a sample. (C) Heat map of transcriptome clustering. Each column represents a sample, each row represents a gene, and the color in the figure indicates the intensity of gene expression in that sample, with red indicating high expression and blue indicating low expression. (Sample names (from left to right) are B250318B, B250306BB, B250220B, B250306B, B250306BBB, B250327B, B250313BB, B250313B, B250320B, B250208B, B250320BBB, B250211B, B250 325B, B250208BB, B250228B, B250220BB, B250320BB, A250211A, A250313AA, B250219B, A250206AAA, A250207A, A241106, A241125, A241120, B241211, A241118, A250103, AAA241211, A250320A, A250207AAAA, A250208AA, A250206A, A250206AA, A250325A, A250207AA, A250208A, A250219A). (D) Principal component analysis plot of the proteome. *E*: Heat map of proteome clustering. (Sample names (from left to right) are B241211, B250211B, B250219B, B250318B, B250320B, B250208B, B250313BB, B250306BBB, B250325B, B250208BB, B250306B, B250327B, B250313B, B250228B, B250320BB, B250320BBB, B250210B, B250306BB, B250220B, B250220BB, A241106, A250206AAA, A250219A, A250207AA, A250207AAAA, A250206A, A250325A, AAA241211, A241125, A250320A, A250211A, A241118, A250206AA, A250207A, A250208A, A250208AA, A241120, A250313AA, A241015, A250103).

A total of 20 keloid tissue samples and 20 matched normal skin samples were obtained from patients undergoing surgical resection (Table 1). Among them, 11 were male and nine were female, aged between 22 and 43 years. There were 13 cases in the Front chest, three cases in the shoulder, three cases in the ear and one case in the abdomen. The score of keloid symptoms in these 20 patients showed that their mVSS index was more than four points but no more than 10 points, and the vast majority of patients were accompanied by mild pain and moderate itching symptoms. Figure 1A illustrates the entire analysis process of the transcriptomic and proteomic differences between the integrated keloid and normal skin in this study.

**Table 1:** Characteristics of patients.

Patients	Gender	Age	Site of disease	Symptom score		
				mVSS (0–13)	Pain (0–10)	Pruritus (0–10)
1	M	34	Front chest	6	2	1
2	M	32	Front chest	6	1	4
3	M	28	Front chest	6	2	6
4	F	22	Shoulder	8	0	3
5	M	40	Front chest	8	2	0
6	F	24	Ear	8	3	3
7	F	38	Front chest	3	0	4
8	F	34	Front chest	8	2	6
9	M	39	Abdomen	7	1	4
10	M	25	Front chest	10	2	6
11	F	31	Ear	7	3	3
12	F	32	Shoulder	4	2	3
13	F	23	Front chest	10	2	1
14	M	23	Front chest	8	1	4
15	F	22	Front chest	10	1	6
16	F	43	Ear	7	0	5
17	M	41	Front chest	10	2	8
18	M	32	Shoulder	9	3	7
19	M	35	Front chest	10	5	8
20	M	28	Front chest	5	1	2

**Note:**

mVSS: The modified Vancouver Scar Scale is used for the descriptive assessment of keloids, including melanin (M, 0–2), height (H, 0–3), vascularity (V, 0–3) and pliability (*P*, 0–5).

### Data quality evaluation

Principal component analysis (PCA) was used to verify the intra-group reproducibility of data between Keloid and Normal skin groups. The analysis results showed that samples between Keloid and Normal groups were significantly separated at the transcriptome level (Fig. 1B), with 49.37% variation in PC1 dimension. Samples from the Keloid group were clearly clustered in the right region on the PC1 axis, while samples from the Normal group were distributed on the left, indicating systematic differences in gene expression profiles between the two groups. In addition, the samples in the two groups were closely distributed, and no obvious outliers were observed, suggesting that the experimental batch effect was well controlled and the data reliability was high. This result further supports that keloid has unique molecular characteristics and provides a basis for subsequent differential gene analysis and pathway enrichment. The heat map of sample hierarchical clustering showed that Keloid and Normal groups samples were significantly separated in the cluster tree (Fig. 1C). Keloid samples were clustered in the lower right branch, and their gene expression profiles showed a high degree of consistency, with little intra-group variation, suggesting that keloid has unique transcriptome characteristics.

Similarly, the results of PCA analysis showed that the samples from the keloid group and the normal group were also separated at the proteome level (Fig. 1D), and the variation in PC1 dimension reached 21.50%. Samples from the keloid group were significantly more left on the PC1 axis than samples from the normal group, indicating systematic differences in protein expression profiles between the two groups. The heat map of the hierarchical clustering of protein samples showed that samples from the keloid group and the normal group were significantly separated in the cluster tree (Fig. 1D). Keloid samples were clustered in the lower right branch, and their protein expression profiles showed a high degree of agreement with little intra-group variation, indicating that keloids have unique proteomic features.

### Identification of differentially expressed genes and proteins

**Figure 2:**
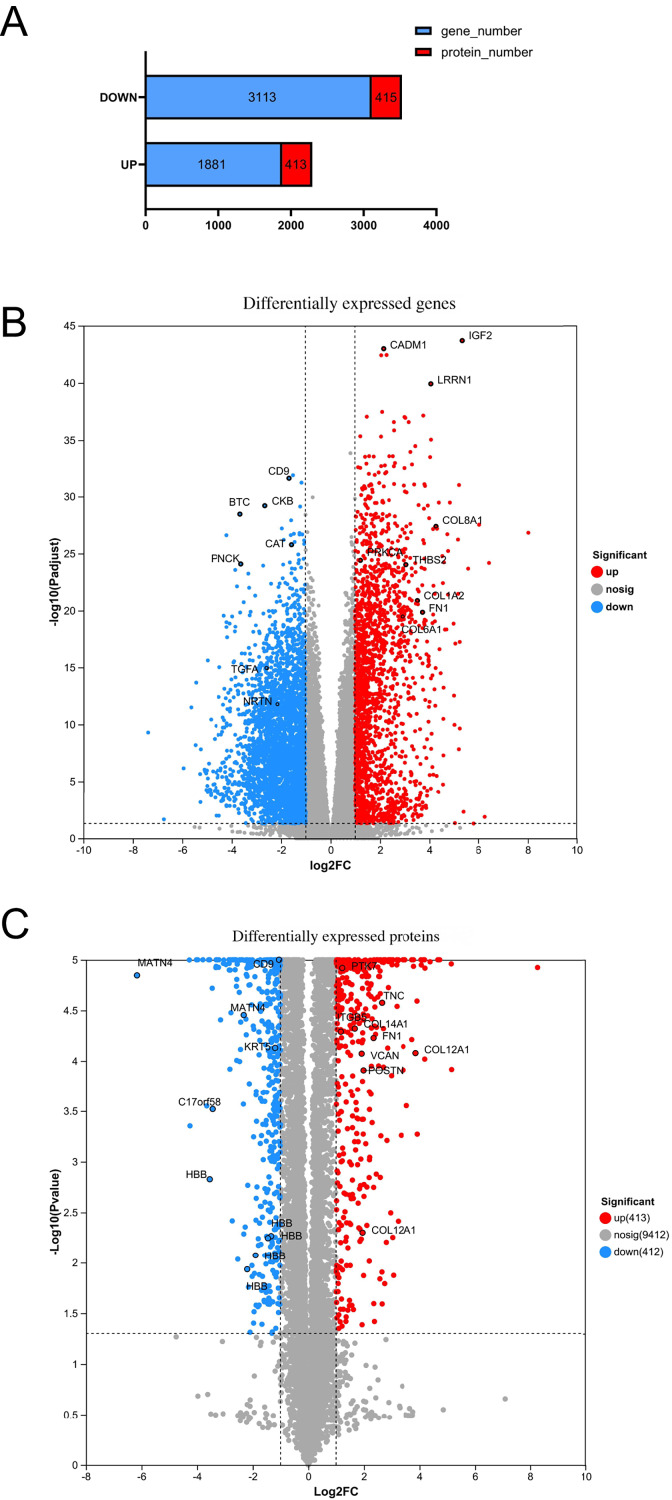
Identification of DEGs and DEPs. (A) Statistical plot of DEGs and DEPs differences. (B) Volcano plot of DEGs. The horizontal axis is the fold change value of the difference in gene expression between the two groups, log-processed. The vertical axis is the statistical test value for the difference in expression of differentially expressed genes, the Padjust value. Red dots indicate significantly up-regulated genes, blue dots indicate significantly down-regulated genes, and gray dots indicate genes that are not significantly differentially expressed. (C) Volcano map of DEPs. The horizontal axis is the fold change value of the difference in gene expression between the two groups, log-processed. The vertical axis is the statistical test value for the difference in expression of differentially expressed proteins, the Padjust value. Red dots indicate significantly up-regulated proteins, blue dots indicate significantly down-regulated proteins, and gray dots indicate proteins that are not significantly differentially expressed. HBB expressed proteins from the HBB family.

Comparison of transcriptomic profiles between keloid and normal tissues identified 4,994 DEGs, comprising 1,881 upregulated and 3,113 downregulated genes (Fig. 2A). Proteomic analysis revealed 828 DEPs, with 413 upregulated and 415 downregulated proteins. Table 2 list the screened differentially expressed genes. Among them, COL11A1, COL12A1, FN1, POSTN, and TNC are significantly upregulated in both the transcriptome and proteome, while MATN4, HBB, and C17orf58 are significantly downregulated. The volcano map was created based on these DEGs and DEPs. In the transcriptome samples of clinical keloids, the levels of several markers of the PI3K-AKT pathway were increased, including insulin-like growth factor 2 (IGF2), protein kinase Cα (PRKCA), thrombopoietin 2 (THBS2), and others. Genes related to extracellular matrix remodeling, such as collagen 8α1, collagen 4α2, collagen 5α1, and fibronectin 1, also showed significant differential expression, indicating a significant increase in collagen deposition in keloids (Figs. 2B, 2C). However, in the proteome samples, the protein levels of ITGB5 and POSTN in the PI3K-AKT/MAPK signaling pathway were significantly increased. Extracellular matrix remodeling-related proteins such as TNC also showed differential expressions. Although BTC, CKB, PNCK and other genes were down-regulated at the transcriptome level, no differential expression was observed at the protein level. Interestingly, HBB family, MATN4 family and C17of58 proteins were significantly decreased in keloid tissues.

**Table 2:** Differentially expressed genes and proteins.

Differentially expressed genes by RNA-seq			Differentially expressed proteins by proteomics		
Gene symbol	Log 2 (fold change)	*P*-value	Protein symbol	Log 2 (fold change)	*P*-value
IGHG1	25	3.61E−35	ETNK2	4.7	6.92E−09
IGF2	24	1.62E−16	COL12A1	3.9	7.34E−05
COL11A1	5.6	2.92E−13	SLC10A7	3.4	3.38E−10
FN1	5.2	6.95E−20	SLC2A13	3.2	1.89E−05
POSTN	5.1	5.41E−09	MAP3K2	2.9	4.48E−11
COL12A1	5.1	2.51E−15	TNC	2.7	1.66E−05
GALNT5	3.8	4.37E−25	FN1	2.3	4.92E−05
TBS2	3.5	1.11E−22	COL11A1	2.3	4.01E−04
TGFB3	3.1	1.47E−19	THBS2	2.3	1.82E−03
CREB3L1	3.0	2.24E−22	COL8A1	2.1	4.23E−03
CDKN2	2.9	1.87E−18	POSTN	2.1	2.06E−05
TNC	2.8	3.33E−23	MATN4	−6.2	4.25E−06
SLC10A3	2.8	2.33E−3	ABCA9	−4.3	4.64E−08
THBS2	2.5	3.98E−23	SPTBN2	−4.3	4.34E−04
TCL1A	2.4	2.67E−06	HBB	−3.5	1.48E−03
MAGED1	2.2	2.25E−36	C17orf58	−3.4	2.92E−04
IGF1	2.1	2.50E−17	KRT66	−2.6	3.37E−08
ORAI2	2.0	1.15E−35	LAMA4	−2.2	2.10E−06
GAREM2	1.9	1.80E−23	KRT19	−1.7	2.74E−04
MAPK12	1.8	7.94E−25	DCN	−1.5	3.82E−04
MATN4	−5.2	3.23E−11	DSP	−1.4	1.62E−04
IL1RN	−4.2	1.26E−03			
KRT31	−3.7	1.73E−14			
KRT23	−3.6	2.44E−17			
IL20RA	−2.9	1.52E−08			
TGFA	−2.6	5.85E−17			
HBB	−2.4	4.75E−07			
SPTBN2	−2.3	1.12E−11			
C17orf58	−1.8	2.96E−07			
ABCA9	−1.3	1.50E−4			

### GO and KEGG pathway enrichment analyses of differentially expressed genes and proteins

**Figure 3:**
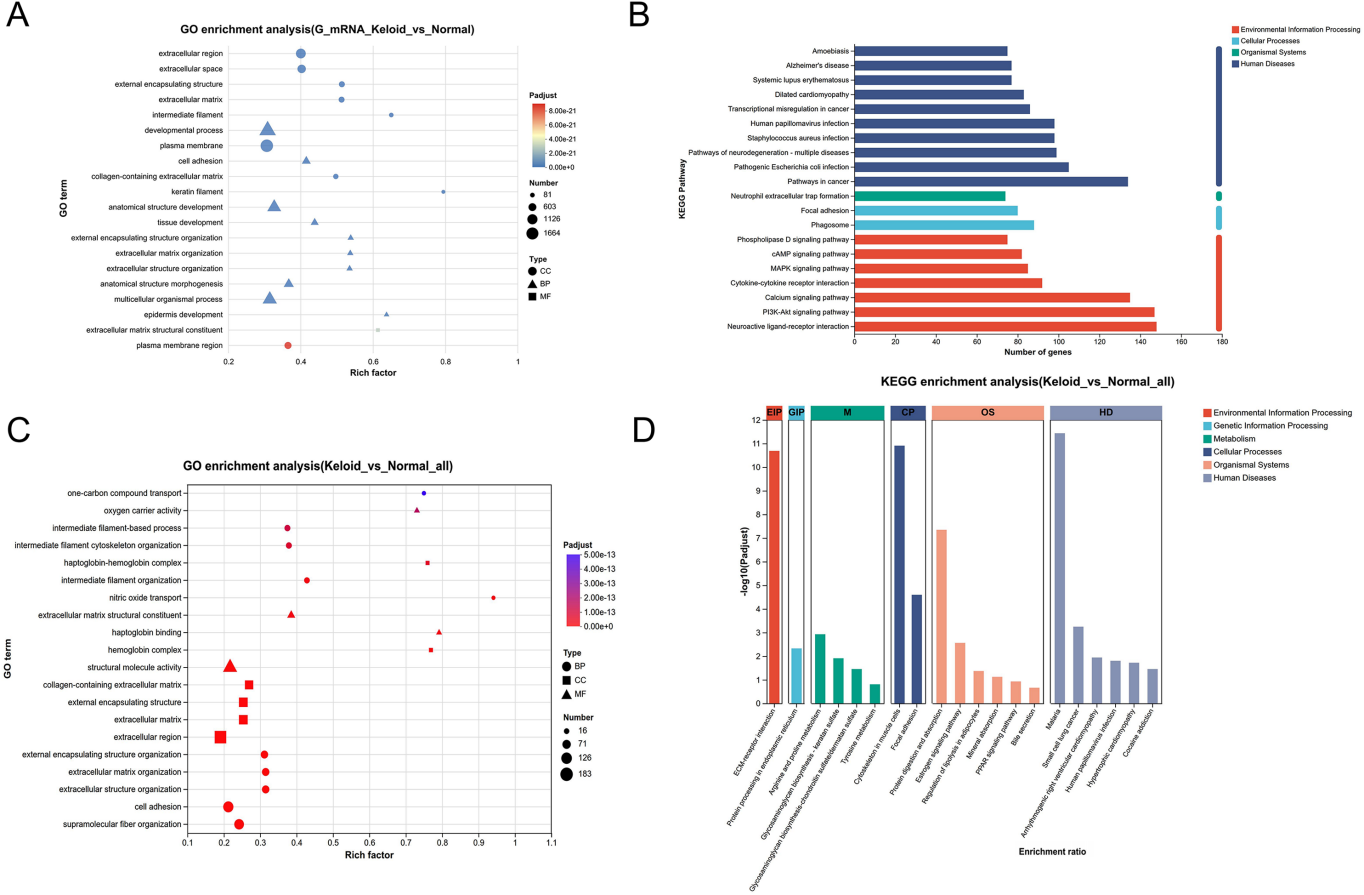
GO and KEGG analysis for DEGs and DEPs. (A) DEGs GO enrichment analysis. Three colors indicate the three major categories, namely biological process (BP), cellular component (CC) and Molecular function (MF); (B) DEGs KEGG enrichment analysis. Different colors indicate the seven branches of KEGG metabolic pathways, namely metabolism (M), genetic information processing (GIP), environmental information processing (EIP), cellular process (CP), Organism system (OS), human disease (HD), and Drug development (DD); (C) DEPs GO enrichment analysis; (D) DEPs KEGG enrichment analysis.

To further analyze the biological functions of DEGs and DEPs in keloids, we performed systematic functional annotation and pathway enrichment analysis of DEGs and DEPs through GO and KEGG databases. GO functional classification statistics showed that DEGs in Keloid group and normal group were mainly concentrated in BP, including extracellular structure organization and developmental process. However, DEPs are mainly significantly enriched in the process of extracellular structure organization and cell adhesion in BP (Figs. 3A, 3B), which is closely related to the invasive growth of Keloid tissue. In the CC dimension, both DEGs and DEPs were mainly located in the extracellular matrix, indicating that pathological extracellular matrix remodeling involves both Keloid gene expression and protein assembly. In MF, DEGs and DEPs were also enriched in structural molecules, which directly confirmed the abnormal genes and pathological construction of ECM components at the protein level. Further KEGG pathway analysis showed that DEGs in Keloid were mainly enriched in PI3K-AKT, mitogen-activated protein kinase (MAPK) signaling pathway, extracellular matrix-receptor interaction and TGF-β signaling pathway, and JAK-STAT signaling pathway (Fig. 3C). DEPs were mainly enriched in ECM-receptor interaction, glycosaminoglycan biosynthesis, cytoskeleton in muscle cells and Focal adhesion pathways (Fig. 3D). These results suggest that both are highly synergistic in the core process and jointly drive the aggressive growth of keloids.

### Hub gene identification

**Figure 4:**
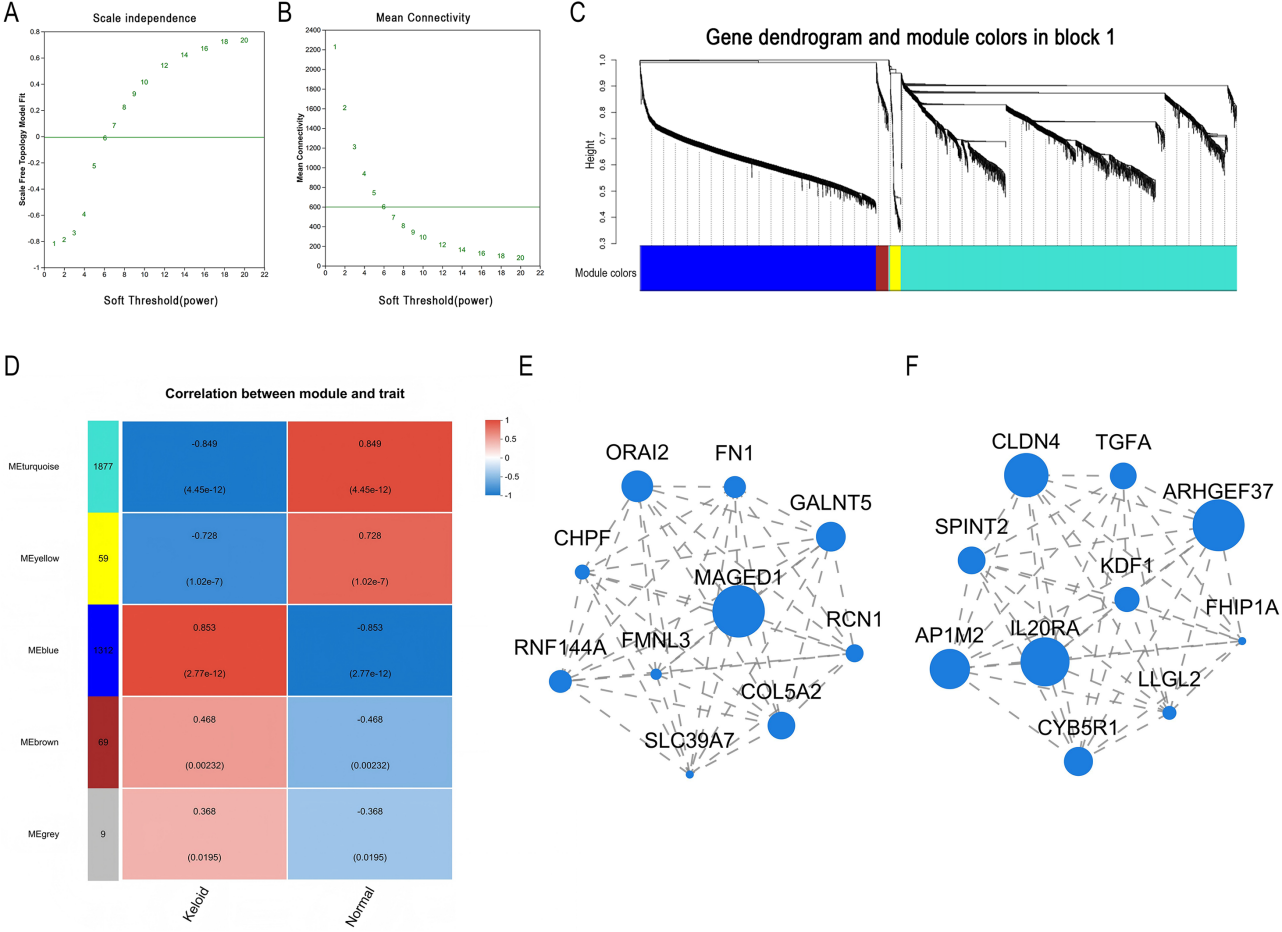
WGCNA was used to identify keloid-related module genes. (A) Scale-free topology fit (left) and mean connectivity curves (right) for optimal soft threshold selection; (B) module clustering dendrogram with horizontal axis colors indicating modules; (C) module-phenotype correlation heatmap: vertical axis modules, horizontal axis groups, colored cells show correlation coefficients (*P*-values)—larger absolute values indicate stronger correlations (blue = negative, red = positive); (D) MEblue module gene co-expression network: nodes represent genes, higher connectivity (more edges) indicates greater importance; (E) MEturquoise module gene co-expression network of significantly up-regulated hub genes in the keloid group. (F) MEturquoise module gene co-expression network of significantly down-regulated hub genes in the keloid group.

Subsequently, WGCNA analysis was performed on all DEGs to construct a gene co-expression network to screen the gene bases closely related to the disease phenotype, as well as the Hub genes within them. A scale-free network (scale-free R^2^ = 0.78) was constructed using a soft power (β value) of 20 (Fig. 4A). WGCNA results clustered the correlation between Keloid and Normal groups traits into five modules, the MEturquoise module clustered to the most 1,877 genes, and the least MEgrey module also had nine genes (Fig. 4B). Among them, the ‘blue’ module (referred to as MEblue) was significantly positively correlated with the Keloid group (r = 0.853, *P* = 2.77 e−12), and the ‘turquoise’ module (referred to as MEturquoise) was significantly negatively correlated with the Keloid group (r = −0.849, *P* = 4.45 e−12) (Fig. 4C). Therefore, we screened the top 10 nodes with connectivity through the MEblue and MEturquoise modules to screen hub genes in the visual network. The results showed that the significantly up-regulated hub genes in the keloid group included MAGED1, ORAI2, GALNT5, COL5A2, FN1 and the significantly down-regulated hub genes included ARHGEF37, IL20RA, CLDN4, TGFA, *etc*. (Figs. 4D–4F).

### PPI network

**Figure 5:**
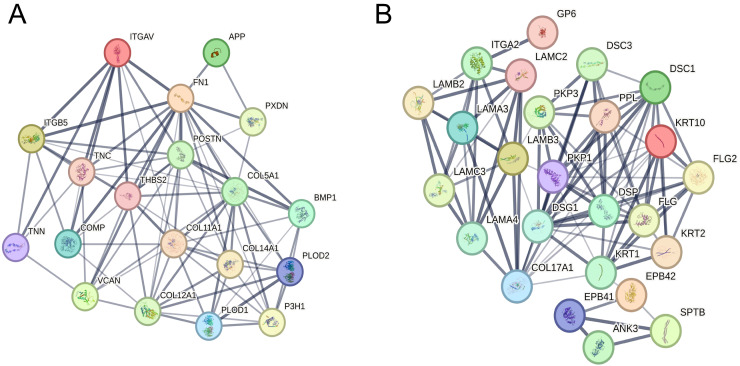
PPI network. (A) PPI network of upregulated DEPs. The edges indicate both functional and physical protein associations, line thickness indicates the strength of data support. (B) PPI network of downregulated DEPs.

A total of 19 significantly up-regulated DEPs and 25 significantly down-regulated DEPs were included in the PPI network, which formed a highly interconnected complex network, respectively, suggesting that these proteins were involved in the pathological process of keloid through synergistic interaction. The core regions of the up-regulated DEPs network were centered on FN1, ITGAV, TNC, THBS2 and collagen family (COL11A1/12A1/14A1, *etc*.) (Fig. 5A), suggesting that the excessive deposition of ECM in keloid is not a single protein abnormality. It is achieved through a multi-molecule cooperative network of ECM synthesis, modification and cell adhesion. The core regions of the down-regulated DEPs network were centered on the keratin family KRT and related proteins (KRT1, KRT2, KRT10, FLG, FLG2), desmosome complex (DSC/DSG/PKP), and basement membrane complex (laminin) (Fig. 5B). It is suggested that the epidermal abnormality of keloid is the result of the destruction of skeletal structure and the disorder of intercellular adhesion.

## Discussion

This study reveals the synergistic pathogenic network of key genes and key pathways in keloid at the molecular level, which provides a new perspective to break through the limitation of single omics.

Through KEGG enrichment analysis, we found that DEGs in keloid were significantly enriched in the PI3K-AKT signaling pathway. The significant up-regulation of IGF2, IGF1, PRKCA and THBS2 at the gene level was complementary to the up-regulation of ITGB5 and POSTN at the protein level. The PI3K-AKT pathway plays a key role in regulating cell survival and proliferation (Fang et al., 2023). Once activated, it can promote fibroblast proliferation and inhibit apoptosis by activating downstream mTOR or inhibiting GSK-3β (Chen et al., 2022; Jere, Houreld & Abrahamse, 2019). Chen et al. (2022) has confirmed that IGF2 and IGF1 are important growth factors, which can activate PI3K-AKT pathway by binding to their receptors. PRKCA, a member of the protein kinase C family, can further enhance the PI3K-AKT signaling cascade when activated (Chen et al., 2024a). Upregulation of MAGED1 modulates the membrane localization of key kinases such as PRKCA, thereby increasing AKT phosphorylation and amplifying proproliferative signals (Wang et al., 2010). These results all indicate that the PI3K-AKT pathway is abnormally activated in keloid fibroblasts, which is positively correlated with the proliferation ability of fibroblasts. Our results are highly consistent with the previous findings of Kong and co-authors, which further confirmed the critical role of PI3K-AKT pathway in keloid formation and development (Kong et al., 2025).

In addition, FN1, COL12A1, COL11A1, TNC, THBS2 and POSTN genes and proteins in the ECM-receptor interaction network were significantly up-regulated at the transcription and translation levels. As a core component of ECM, FN1 not only participates in tissue remodeling, but also activates the PI3K-AKT pathway through integrin-mediated adhesion signaling (Livingston et al., 2023). The regulation of downstream mTOR signaling drives cell cycle progression, forms a positive feedback loop, and aggravates the pathological process of fibroblasts. This mode of multi-molecule collaboration also explains the limited efficacy of traditional single-target therapies, such as TGF-β inhibitors (Zhang et al., 2020). COL12A1 and COL11A1 are involved in collagen synthesis (Feng et al., 2022), TNC and THBS2 participate in cell-matrix adhesion and ECM assembly (Chen et al., 2024b), and POSTN is also closely related to tissue repair and fibrosis (Wu et al., 2022). The upregulation of these genes and proteins strengthens the interaction between fibroblasts and ECM, promoting ECM deposition and excessive fibrotic growth of keloids.

In the MAPK pathway, MAPK12 and GAREM2 were up-regulated in the transcriptome and MAP3K2 in the proteome, and the calcium signal regulator ORAI2 (WGCNA hub gene) formed a migration regulatory network. Wu et al. (2021) found that ORAI2 activates MAPK/ERK by mediating calcium influx and promotes fibroblast migration. Snow et al. (2014), Karin, Liu & Zandi (1997) further revealed that aberrant calcium signaling can activate transcription factors such as NF-κB and AP-1 and induce the expression of proinflammatory cytokines and fibrosis-related genes. Notably, upregulation of GAREM2 as a MAPK scaffold protein also enhanced ERK signaling specifically to pro-migratory transcription factors such as AP-1. This is highly consistent with the “cell migration” and “matrix degradation” functions enriched in GO, suggesting that MAPK pathway mediates the invasive phenotype of fibroblast “tumor-like” in keloid.

The hub gene interleukin-20 receptor alpha (IL20RA) is a key molecular feature of cytokine pathway abnormalities (Liu et al., 2022a). IL-20 can inhibit TGF-β-induced COL1A1 expression through the JAK-STAT pathway, and its receptor IL20RA mainly plays an anti-inflammatory and tissue repair role in normal skin tissue (Gao et al., 2021). Chiriac M T can alleviate the negative regulation of fibrosis by inhibiting the expression of IL20RA, aggravate the infiltration of M2 macrophages and the secretion of pro-inflammatory cytokines (such as TNF-α and IL-6), leading to the formation of a pro-inflammatory immune microenvironment (Chiriac et al., 2024). Therefore, downregulation of IL20RA and TGFA makes proinflammatory signaling dominant in keloids.

The expression of KRT family genes KRT31 and KRT23 were down-regulated at the transcriptional level, KRT19 was down-regulated at the protein level, and the desmosome proteins DSG/DSC were also down-regulated. Keratins are essential components of the epidermal cytoskeleton, providing mechanical support and maintaining cell integrity (Klein et al., 2024). Downregulation of KRT family genes and desmosome proteins leads to disruption of the epidermal cytoskeleton and affects the normal structure and function of the epidermis (Chitaev et al., 1996; Buchroithner et al., 2021). Therefore, the abnormal epidermis of keloid may be the result of the destruction of skeleton structure and the disorder of intercellular adhesion.

The expression of SLC10A3 gene is up-regulated in keloid patients, and the expression of SLC10A7 and SLC2A13 proteins is up-regulated, suggesting the imbalance of microenvironment homeostasis. The SLC gene family is involved in the transport of various substances such as ions and nutrients (Le et al., 2024). The abnormal expression of multiple genes and proteins can affect the transport and homeostasis of related substances in keloid tissue, thereby affecting cell metabolism and function. For example, SLC10A3 is involved in bile acid transport, and its upregulation may affect bile acid metabolism in keloid tissue, which in turn affects cell signaling and inflammatory responses (Claro da Silva, Polli & Swaan, 2013). SLC2A13 functions as a hypoxia-induced glucose transporter and may supply fibrotic energy demand by accelerating glycolysis (Gou et al., 2023). The up-regulation of SLC10A7 may activate FXR nuclear receptor and indirectly regulate TGF-β signaling (Kersseboom et al., 2017), which has been confirmed in liver fibrosis, but is the first report in keloid.

The expressions of HBB family and C17orf58 genes and proteins were also significantly down-regulated in keloid tissues. Hemoglobin is mainly involved in oxygen transport and plays a role in REDOX regulation and cell signaling (Gell, 2018). The down-regulation of HBB family genes and proteins suggests that there is a hypoxic microenvironment in keloid, which may be related to its abnormal REDOX state. Xu et al. (2024) demonstrated in lung cancer tissue and plasma samples that HBB can regulate cell proliferation through the ERK1/2 pathway and play a key role in the tumorigenesis and progression of the disease. In addition, the hypoxic environment of keloid can induce the production of ROS, and then activate pro-fibrotic signaling pathways such as NF-κB (Wang et al., 2021), but its specific role in the pathogenesis needs to be further explored.

In summary, the hub genes screened in this study show significant functional clusters. The up—regulated genes, such as MAGED1, FN1 and COL5A2, are mainly enriched in ECM remodeling, PI3K-AKT/MAPK signaling, and cell adhesion and migration pathways. The down-regulated genes, such as CLDN4, ARHGEF37, IL20RA and AP1M2, are closely related to tight junctions and immune regulation pathways. This bidirectional regulatory network reveals the core pathological mechanisms that promote abnormal fibroblast proliferation and fibrosis in keloids. These genes not only constitute the molecular nodes of multiple pathways but also provide precise intervention sites for the development of targeted drugs.

Although this study has systematically analyzed the transcriptome and proteome characteristics of keloid, there are still the following limitations. First, the sample size of this study is small, and the cohort needs to be expanded to verify the clinical relevance of Hub genes. Second, studies lacked functional experiments to verify the direct effects of genes on fibroblast phenotypes. In the future, subsequent studies will focus on establishing the causality of these hub genes through functional experiments *in vitro* and *in vivo*, and on evaluating the therapeutic potential of targeting the PI3K-AKT/MAPK pathways in preclinical models.

## Conclusion

Through multi-omics integration, this study identified PI3K-AKT, MAPK, TGF-β, and JAK-STAT pathways as core regulatory networks in keloids. Key upregulated Hub genes (MAGED1, FN1, COL5A2) drive ECM overdeposition and cell adhesion, and downregulated genes (keratin family, desmosome proteins) disrupting epidermal structure and barrier function. These findings link overactivation of fibroblasts to epidermal dysfunction, providing a rationale for the development of multitargeted therapies targeting cell signaling and epidermal repair. In the future, the sample cohort will be expanded, the function of Hub genes will be verified by *in vitro* experiments, and small molecule inhibitors targeting different pathways will be explored in animal models to advance the practical application of these therapies.

## Additional Information and Declarations

### Data Availability

The following information was supplied regarding data availability:

The raw data is available at the China National Center for Bioinformation: OMIX012709.
